# The current MLVA typing scheme for *Enterococcus faecium *is less discriminatory than MLST and PFGE for epidemic-virulent, hospital-adapted clonal types

**DOI:** 10.1186/1471-2180-7-28

**Published:** 2007-04-10

**Authors:** Guido Werner, Ingo Klare, Wolfgang Witte

**Affiliations:** 1 Robert Koch Institute, Wernigerode Branch, Wernigerode, Germany

## Abstract

**Background:**

MLVA (multiple-locus variable-number tandem repeat analysis) is a reliable typing technique introduced recently to differentiate also isolates of *Enterococcus faecium*. We used the established VNTR (variable number of tandem repeats) scheme to test its suitability to differentiate 58 *E. faecium *isolates representing mainly outbreaks and clusters of infections and colonizations among patients from 31 German hospitals. All isolates were vancomycin-resistant (*vanA *type). Typing results for MLVA are compared with results of macrorestriction analysis in PFGE (pulsed-field gel electrophoresis) and MLST (multi-locus sequence typing).

**Results:**

All 51 but one hospital isolates from 1996–2006 were assigned to the clonal complex (CC) of epidemic-virulent, hospital-adapted lineages (MLST CC-17; MLVA CC-1) and differed from isolates of sporadic infections and colonizations (n = 7; 1991–1995) and other non-hospital origins (n = 27). Typing of all 58 hospital VRE revealed MLVA as the least discriminatory method (Simpson's diversity index 0.847) when compared to MLST (0.911) and PFGE (0.976). The two most common MLVA types MT-1 (n = 16) and MT-159 (n = 14) combined isolates of several MLST types including also major epidemic, hospital-adapted, clonal types (MT-1: ST-17, ST-18, ST-280, ST-282; MT-159: ST-78, ST-192, ST-203). These data clearly indicate that non-related *E. faecium *could possess an identical MLVA type being especially critical when MLVA is used to elucidate supposed outbreaks with *E. faecium *within a single or among different hospitals. Stability of a given MLVA profile MT-12 (ST-117) during an outbreak over a period of five years was also shown.

**Conclusion:**

MLVA is a suitable method to assign isolates of *E. faecium *into distinct clonal complexes. To investigate outbreaks the current MLVA typing scheme for *E. faecium *does not discriminate enough and cannot be recommended as a standard superior to PFGE.

## Background

Effective typing of microorganisms is a prerequisite for establishing epidemiological or phylogenetic links between corresponding isolates. A plethora of different methods has been successfully applied to type and differentiate bacterial strains and clonal groups from each other [1]. A critical point to all of these methods is their applicability to answer distinct questions ranging from investigation of outbreaks to establishing rather broad phylogenetic trees of relatedness and arrangement of strains within major clonal complexes. Each method has its respective weaknesses and strengths according to the question(s) addressed and the methodology behind [2-7].

Recently, a new method was introduced using small repetitive elements appearing in a variable number and distributed among the genome of a given species. Accordingly this technique based on a variable number of tandem repeats (VNTR) was named multiple-locus variable-number tandem repeat analysis (MLVA; [8]). Initially MLVA was established to differentiate high-risk pathogens such as *Bacillus anthracis *and *Francisella tularensis *[9-11] but has been extended to a numerous number of other bacterial species and scientific questions [8,12] including outbreak investigations for pathogenic bacteria [4,13].

MLVA was applied also recently to type isolates of *Enterococcus *spp. Its discriminatory power was compared to MLST for a collection of 392 *E. faecium *[14] and to macrorestriction analysis in PFGE for 83 *E. faecalis *[15]. In both cases it was described that MLVA showed similar and rather concordant discrimination when compared to the respective reference method. Although the selected VNTRs were different between the two species, the overall conclusion would suggest MLVA as a typing method on one hand to discriminate highly enough between strains and on the other hand indicate the possibility to establish rather broad phylogenetic relatednesses. To support this hypotheses and to test the applicability of the established MLVA scheme for *E. faecium *to indicate and differentiate hospital-adapted clonal types appearing in increasing numbers among hospital patients worldwide [16], we investigated hospital isolates representing outbreaks and clusters of infections and colonizations from German hospital patients from the last 15 years using MLVA, *Sma*I-macrorestriction analysis in PFGE, and MLST. A collection of 27 *E. faecium *from commensal, animal, and environmental origins was included for reasons of comparison and also typed by MLVA.

## Results

### Macrorestriction analysis in PFGE

**Figure 1:**
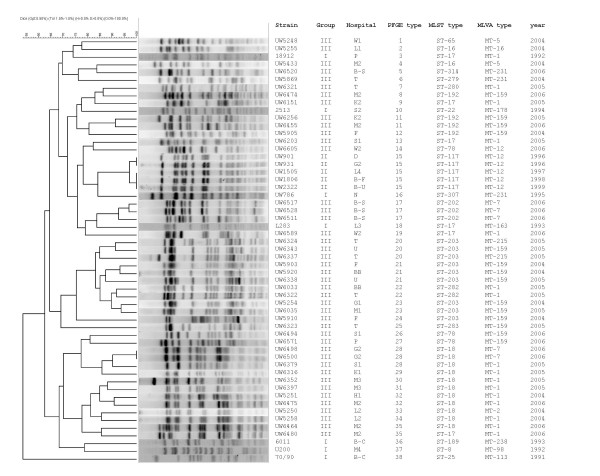
**Cluster analysis of hospital VRE based on *Sma*I-macrorestriction patterns resolved in PFGE **(see also Tab. 1). The dendrogram was evaluated using Dice coefficient and UPMGA clustering (BioNumercis software). Legend: Groups – group I isolates from 1991–1995, group II isolates from 1996–1999, group III isolates from 2004–2006; year, year of isolation; hospital, code for hospital. Only five of all eight group II isolates were shown having all identical or highly related banding patterns (> 95 % identity; see also [30])

Altogether 58 *E. faecium *were investigated by *Sma*I-macrorestriction analysis originating from 31 German hospitals. Thirty-eight different PFGE types were assigned based on a 90 % similarity cut-off and recommendations described recently [17,18]. However, larger clusters of strains possessing at least related patterns (> 80 % identity) could be identified ([18]; see below and Fig. 1).

### MLST

Altogether 19 MLST types were assigned. All but one hospital-adapted/outbreak isolates (n = 50/51) of groups II (1996–1999) and III (2004–2006) possessed MLST types (ST) which belong to MLST CC-17 or C1 representing epidemic and hospital-adapted clonal types (Table 1). Isolate UW6520 revealed ST-314 which belongs to MLST cluster C and not to cluster C1/CC17. Isolates of group I (1991–1995) were quite diverse; only three possessed a MLST type belonging to MLST CC17 (ST-17), the other four were attributed to three different clonal complexes.

### MLVA

Altogether 14 MLVA types were assigned. All 51 group II and III isolates possessed MLVA types associated with clonal complex C1 of hospital-adapted types (Table 1). Cluster assignments of new MLVA types were done by the curator of the MLVA internet pages (J. Top, University Utrecht, Netherlands) based on pairwise similarities, UPMGA clustering and similarities within a minimum spanning tree of relatedness. Four group I VRE belonged to MLVA clonal complex C1, the other three each one to A, B or C (Table 1, see also [14]). The 27 isolates of different non-hospital origins showed ten MLVA types. All but two belonged to MLVA clonal complexes A or B (J. Top, pers. communication). Main type was MT-89 (n = 9; CC-A). Six isolates were none-typeable due to an incapability of an amplification of one of the six VNTR fragments.

### Concordance of PFGE, MLVA and MLST results

Altogether 58 hospital VRE (groups I – III) were typed by all three molecular typing methods. *Sma*I-macrorestriction in PFGE revealed 38, MLST 19, and MLVA 14 different types. PFGE was the most discriminatory method with a discriminatory index of 0.972, MLST the median (0.91) and MLVA the least discriminatory (0.842) (Table 2). Differences in discriminatory power between those different typing methods are even more obvious and pronounced when results involving only outbreak isolates of groups II and III are compared (PFGE: 0.965; MLST: 0.891; MLVA: 0.814). MLVA appears in all cases as the least discriminatory method which derives from the fact that (i) it revealed the least number of different types and in addition, (ii) more than half of all the investigated VRE belong to only two types MT-1 and MT-159.

**Figure 2:**
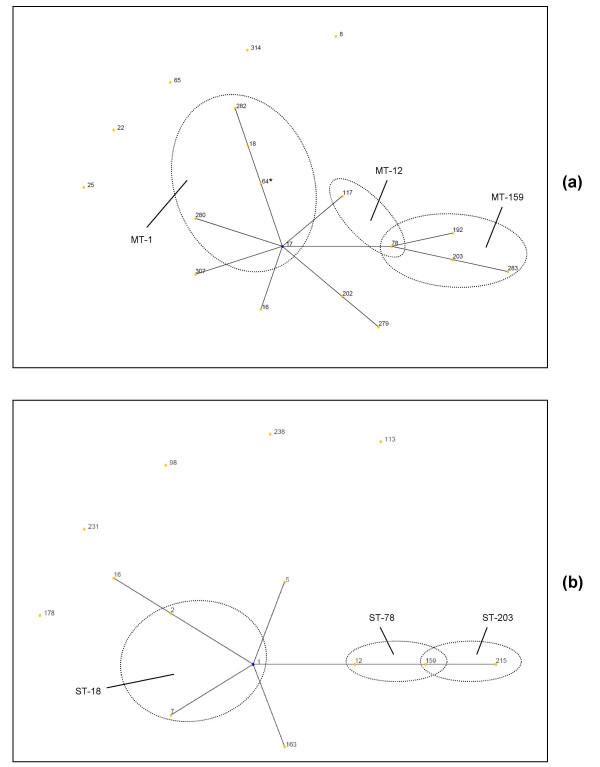
**Comparison of phylogenetic relatedness between identified MLST (a) and MLVA types (b) for all 58 hospital VRE**. Corresponding trees using eBURST v3 showing clades of an identical MLVA type (MT) for several MLST types (ST) (a) and an identical ST for several MTs (b), respectively. Each line separates MLVA/MLST types differing by a single locus (single locus variants). ST-64 is not part of the given collection and was included for a better illustration of relatedness between MLST types. The MLVA type of ST-64 is not known.

Some isolates with identical MLVA types showed different MLST types (Table 1; Fig. 2). But also the opposite was found; isolates with identical MLST types could possess different MLVA types (Table 1; Fig. 2). Isolates harboring the two major MLVA types, MT-1 and MT-159, combine isolates showing a various number of STs. E.g., MT-159 isolates combine *E. faecium *of ST-78, -192, -203, and -283. MT-1 isolates combine *E. faecium *of ST-17, ST-18, ST-280, and ST-282.

Results of MLST partly confirm cluster assignments based on PFGE data, whereas our data did not show a visible concordance between MLVA and PFGE (Fig. 1). Many MLVA types were distributed among different main PFGE clusters. Especially the most common types such as MT-1 (n = 16) and MT-159 (n = 14) were evenly distributed among different major PFGE branches. This fact is also reflected in the much lower concordance value for results of MLVA/PFGE (0.863) compared to MLST/PFGE (0.935; Table 2). Eight of nine ST-203 isolates cluster in a group of related PFGE patterns (> 83 % identity) together with two ST-282 VRE (ST-282 is a double-locus variant [DLV] of ST-203). A single ST-203 isolate clusters in a side branch but at least within a cluster of > 75 % identity. These eleven isolates possess three different MLVA types. All eleven ST-18 *E. faecium *cluster in a group with > 70 % identical patterns. However, also two ST-78 and a single ST-17 isolates belong to this cluster but are located at both edges of this branch. These altogether 14 VRE possess four MLVA types. In contrast to this, isolates of ST-17 appear distributed among different branches of the PFGE tree.

The eight group II isolates all possess an identical MLST (ST-117) and MLVA (MT-12) type and an identical or highly related PFGE pattern (> 95 % identity; only shown for five isolates). Isolate UW6605 (ST-78) revealed also MT-12 but is clearly different from group II isolates when compared on the basis of its PFGE pattern. However, ST-117 and ST-78 might be phylogenetically related since both MLST types only differ in a single locus.

## Discussion

MLVA was introduced to molecular bacterial strain typing as a promising alternative to established typing standard methods such as AFLP, MLST and partly PFGE or as an alternative for microorganisms where other common typing techniques could not be applied due to several reasons [19]. Therefore it was not possible in all cases to compare its discriminatory power to established standards which is expected to vary also from genus to genus. For nosocomial pathogens, such as *Staphylococcus aureus*, MLVA was introduced as an alternative typing method showing matching results with macrorestriction typing in PFGE and clusters generated by *spa-*typing and MLST [4]. For *E. faecium *discriminatory power of MLVA was compared to MLST and AFLP (Amplified fragment length polymorphism) and found to be reliably sufficient as a suitable typing alternative [14]. But already when comparing data from its original description in detail, some uncertainties emerged challenging the overall applicability of this method for typing in general including especially investigation of outbreaks or following routes of disseminated outbreak strains. For instance, MT-1 combined 58 isolates represented by eleven STs [14]. Since MT-1 is the primary founder and one of the major MLVA types representing the cluster of hospital-adapted, clonal types (MLVA C1) its applicability to investigate single outbreak situations could be questioned. Indeed, this was one of the results of our study: Isolates representing several MTs such as the most common MT-1 and MT-159 neither cluster based on their *Sma*I-macrorestriction patterns in PFGE nor possess all identical or related MLST types (Fig. 1 and 2).

As shown by our results, discrimination of *E. faecium *based on results of MLVA, MLST and PFGE does not always give concordant results as it was shown for rather clonal populations such as *S. aureus *[7] or for an analysis of *E. faecium *within a single hospital over a period of five years [20]. Concerning the latter a more detailed comparison of the results of Abele-Horn et al., and ours may be required. These, at the first moment rather contradictory results reflect major differences in the used strain collection (strains from a single hospital vs. 31 hospitals) and correspondingly resulting MLVA and MLST type diversity. A collection of isolates from a single hospital may not be diverse enough to elucidate the non-congruencies we found here between some MLVA and MLST types (e.g., MT-159 absent in [20]). The discriminatory power of MLVA in strains recovered from patients at the University Hospital in general, i.e. not only associated with the described outbreaks, revealed a Simpson's diversity index of 0.846 [20] and was almost identical to what we calculated for our study population (0.842).

Clonal types might be affected by genomic rearrangements to a different extent. Whereas ST-18 or ST-203 isolates showed rather similar *Sma*I-macrorestriction patterns over time and geographical distribution, isolates of ST-17 were rather diverse. The ST-17 isolates were distributed among several subclusters or represent single PFGE patterns (Fig. 1). It could be speculated if isolates of ST-17 represent a rather ancient clonal type prone to recombination and thus divergent PFGE patterns for a longer time than ST-203 and ST-18 which could constitute rather recent hospital-adapted clones. Investigation of ST-17 from the early 1990ies as shown in our small collection of group I isolates and in a collection of bacteremia isolates from the mid to the late 1990ies (Werner, unpublished results) supports this hypothesis. The role of mobile and conjugative elements in rearranging bacterial genomes is well-known [21]. The genome sequence of *E. faecalis *V583 revealed one of the highest numbers of mobile, integrative and conjugative elements known so far from the bacterial world [22]. Similar numbers are suggested for *E. faecium*, however whole genome data for *E. faecium *are still incomplete. Recent comparative genomic hybridizations for a set of 97 *E. faecium *from different origins confirm the role of mobile and conjugative elements for shaping the *E. faecium *genome [23]. IS element driven recombination appeared higher in a subset of evolutionary linked epidemic *E. faecium *than in non-epidemic *E. faecium *(based on a comparison of typing results for MLST and PFGE). In this respect PFGE may be "overdiscriminatory" to a certain extent when applied to investigate so-called hospital clade or epidemic *E. faecium *[23]. Other factors and impacts that affect distinct clonal types in a different manner could also be discussed [24,25].

Clearly, MLVA has its advantages when applied to type *E. faecium*. It is a suitable method to identify strains belonging to the complex of hospital-adapted, epidemic clones (MLVA CC-1). The method is quick (intra-day results), easy to perform, comparably cheap, with excellent reproducibility (intra- and inter-laboratory) and allows data transfer and comparison. The comparably long repeat lengths of the VNTR loci used to type *E. faecium *make the assay suitable for agarose gel separation and therefore capable for many standard laboratories. Introduction of fluorescence-labelled primers in future may lead to a combined approach of six single reactions into one or two multiplex PCRs [26].

However, the advantage of comparably long repeat lengths may lead to an unintentional side-effect. MLVA was primarily established as a typing method to differentiate between strains. The VNTR repeats are, in general, very short and in a range of 6 to 30 bp [11,12,27]. The idea behind MLVA typing is mainly based on errors caused by the DNA polymerase resulting in slippage and/or recombination in a somehow expectable manner within these sequences. It could be speculated if repeat lengths of the sizes used to type isolates of *E. faecium *(120 – 279 bp) may be too long to let these events appear in an essential frequency. So differences between strains would be less detected and the identified changes would only be suitable to establish phylogenetic links between strain types similar to or even less discriminatory than MLST. Indeed, some VNTR fragments showed length of incomplete repeats suggesting recombinational events within the distinct single repeat units. Assignment of VNTR repeat numbers might be rather erroneous in those cases. Analysis of tandem-repeat fragment length was recently reported to provide misleading results about the phylogeny and subspecies affiliation of *Francisella tularensis *isolates. This was found to be due to non-homologous, sequence-different VNTR fragments of identical lengths in unrelated isolates [28]. It should be emphasized again that the models predicting stability and flexibility of the distinct genomic repetitive structures used for VNTR analysis in *E. faecium *and other bacteria are rather theoretical and speculative. Nothing is really known about the conditions affecting stability of these structures. Due to this uncertainty, establishing phylogenetic relatedness in appropriate trees by a link via single-locus or double-locus variants of MLVA profiles which might suggest an evolutionary link should be interpreted with caution.

## Conclusion

Altogether 58 *E. faecium *mainly from clusters of infections and colonizations and outbreaks among patients from 31 German hospitals were typed by MLVA. Results were compared to results of PFGE and MLST which led to the following conclusions: (i) MLVA is a suitable method to identify isolates of epidemic-virulent *E. faecium *clonal lineages and differentiate them from isolates of other origins; (ii) for isolates of the clonal complex of hospital-adapted strains MLVA is the least discriminatory method when compared to MLST and PFGE and MLVA results are less concordant than MLST results when compared to results of PFGE; and thus (iii) MLVA appears not to be suitable to identify strain types of *E. faecium *in general, e.g. as necessary in cases of outbreaks. The data presented here for *E. faecium *clearly indicate that non-related isolates could possess an identical MLVA type. This would lead to false-positive results being especially critical when MLVA is used to elucidate supposed outbreaks with *E. faecium *within a single or among different hospitals.

## Methods

### Bacterial strains

Majority of isolates (n = 43) originated from recent outbreaks and clusters of infections and colonizations (CCI) in German hospital patients between 2004 and 2006 (group III). Each isolate represents a single case/patient. We stick to the term CCI since outbreak means cases of infections. Our sample collection also includes cases of colonizations (stool samples) and cases of infections where the role of *E. faecium *is not clear (pneumonia, UTI). According to common outbreak definitions, a cluster is represented by n ≥ 2 cases which were epidemiologically linked. Isolates UW5248-UW5258 (n = 6) originated from a large private laboratory service provider in Southwestern Germany with a representative coverage of hospitals in five federal states (Labor Limbach, Heidelberg, Germany). Six representative isolates were chosen from a larger set of isolates representing clusters in five hospitals [29]. The other 37 group III *E. faecium *originated from 14 other German hospitals. Additional eight isolates representing each one an older outbreak sampled all over Germany between 1996–1999 were also included (group II; [30]). All seven but one (UW786) older hospital isolates represent sporadic infections or colonizations in hospital patients between 1991–1995 (group I; [31]). All hospital *E. faecium *were vancomycin-resistant and possessed the *vanA *gene cluster. For reasons of comparison 27 *E. faecium *isolates from non-hospital origins as well as the *vanA *reference strain BM4147 were also included [31,32].

### DNA isolation

DNA was isolated by a column-based technique as recommended by the manufacturer (DNeasy Tissue Kit, Qiagen, Hilden, Germany). Standard protocol was slightly modified starting with an initial cell wall lysis step of 1 ml overnight culture centrifuged and resuspended in 400 μl TES buffer (10 mM Tris, 1 mM EDTA, 10 % saccharose) plus 10 mg/ml lysozyme and incubated at 37°C for 30 min.

*Macrorestriction analysis in PFGE*. Genomic DNA was isolated, digested with restriction endonuclease *Sma*I, and treated as described recently [29]. The agarose gel concentration was 1 %, the CHEF-DR III apparatus (BIO-RAD laboratories, Hercules, CA, USA) was used for PFGE. The ramped pulsed times were as follows: 1–11 sec for 15 h and 11–30 sec for 14 h at 14°C. Relatedness between banding patterns was calculated using a band based similarity coefficient (Dice) and UPMGA clustering (BioNumerics, Applied Maths, Sint-Martens-Latem, Belgium). A composite tree of all 58 hospital VRE of groups I – III resolved in 18 independent PFGE gels was generated the same way with the exception of increasing the value of the "position tolerance setting" to 1.5 % (default: position tolerance setting 1.0 %; optimization 0.5 %). PFGE types were assigned based on >90 % similar patterns and additionally considering recommendations for fragment pattern analyses as described recently [17,18].

### MLST

PCRs amplifying the seven loci used for MLST were done according to the reference [33]. Sequencing reactions were performed according to the manufacturer's recommendations for cycle sequencing of PCR products (Applied Biosystems Germany, Darmstadt). Sequence files were read, evaluated, aligned, and compared to the reference set of alleles using sequencing software Lasergene 6.1. from DNA-STAR (SeqMan 6.1; EditSeq 6.1), TraceEditPro from Ridom [34], and via the official MLST webpage [33]. Assignment of new MLST types and allocation to their corresponding clonal complexes was done by the curator of the internet service (Dr. R. J. L. Willems, University Utrecht, The Netherlands).

### MLVA

MLVA VNTRs were amplified according to Top et al., [14] using modifications given at the corresponding website of the University of Utrecht, The Netherlands [35]. Pure and high-yield genomic DNA isolated via commercially available kits is especially essential for a successful amplification of VNTR-2. MLVA types were assigned using the corresponding tool at the webpage (see above). For unknown profiles a new MLVA type was assigned by the operator of the internet service (Mrs. J. Top, University Utrecht, The Netherlands).

### Phylogenetic analyses

Software eBURST v3 was used to establish trees of phylogenetic relatedness between the isolates based on their MLST or MLVA profiles [36]. Significance of branching of the calculated trees was evaluated by bootstrap analysis of 1000 computer-generated trees. Clonal complexes are defined as described recently [14,16]. Discriminatory indices for and level of congruence between results of MLST, MLVA and PFGE were calculated using Ridom software (Ridom Bioinformatics, Muenster, Germany) based on references given [37-39]. However, the corresponding values should not be interpreted as a stand-alone result. Calculating similarity indices and congruence between methods requires sample collections that are rather unlinked. Our study strain collection is admittedly biased since it involves only hospital *E. faecium*. So we recommend taking this into consideration and comparing those values only within our study strain collection.

## Abbreviations

DLV – double locus variant; MLST – Multi-locus sequence typing; MLVA – multiple-locus variable-number tandem repeat analysis; PFGE – pulsed-field gel electrophoresis; VNTR – variable number of tandem repeat.

## Authors' contributions

GW performed the MLVA typing and cluster analysis, parts of the MLST sequencing and analyses, analyzed macrorestriction results and wrote the manuscript. IK supervised the strain collection including establishing the main data set (contributor's data, resistance profile, standard PCRs, etc.), assorted and allocated isolates for PFGE analyses, and did parts of the MLST sequencing and analyses. WW supervised the study, established contacts to our main contributors of strains, supervised assortment of isolates and critically read the manuscript. All authors read and approved the final manuscript.

## Figures and Tables

**Table 1:** Characteristics of investigated *E. faecium*.

**Isolate**	**VRE**	**Origin**	**Hospital/**	**year**	**PFGE**	**MLST**	**MLST**	**MLST**	**VNTR**^1^	**MLVA**	**MLVA**	**Reference**
	***vanA***		**Town**		**type**	**type**	**alleles**	**CC**		**type**	**CC**	
**Group III**												

UW5255	**+**	CCI	L1	2004	2	16	1-2-1-1-1-1-1	C1/17	5-6-3-2-2-3	16	C1	this study
UW5250	**+**	CCI	L2	2004	33	18	7-1-1-1-5-1-1	C1/17	5-6-3-3-2-3	2	C1	this study
UW5258	**+**	CCI	L2	2004	34	18	7-1-1-1-5-1-1	C1/17	5-7-3-3-2-3	1	C1	[29]
UW5248	**+**	CCI	W1	2004	1	65	1-2-1-20-1-1-9	C1/17	5-7-3-2-2-3	5	C1	[29]
UW5254	**+**	CCI	G1	2004	23	203	15-1-1-1-1-20-1	C1/17	5-7-3-3-1-2	159	C1	[29]
UW5251	**+**	CCI	H1	2004	32	18	7-1-1-1-5-1-1	C1/17	5-7-3-3-2-3	1	C1	[29]
UW5920	**+**	CCI	BB	2004	21	203	15-1-1-1-1-20-1	C1/17	5-7-3-3-1-2	159	C1	[29]
UW6033	**+**	B/CCI	BB	2005	22	282	7-1-1-1-5-20-1	C1/17	5-7-3-3-2-3	1	C1	this study
UW5905	**+**	B/CCI	F	2004	12	192	15-1-1-1-1-7-1	C1/17	5-7-3-3-1-2	159	C1	[29]
UW5903	**+**	CCI	F	2004	21	203	15-1-1-1-1-20-1	C1/17	5-7-3-3-1-2	159	C1	[29]
UW5910	**+**	CCI	F	2004	24	203	15-1-1-1-1-20-1	C1/17	5-7-3-3-1-2	159	C1	[29]
UW6316	**+**	CCI	K1	2005	29	18	7-1-1-1-5-1-1	C1/17	5-7-3-3-2-3	1	C1	this study
UW6035	**+**	CCI	M1	2005	23	203	15-1-1-1-1-20-1	C1/17	5-7-3-3-1-2	159	C1	[29]
UW5433	**+**	CCI	M2	2004	4	16	1-2-1-1-1-1-1	C1/17	5-7-3-2-2-3	5	C1	[29]
UW6480	**+**	CCI	M2	2006	35	17	1-1-1-1-1-1-1	C1/17	5-7-3-3-2-3	1	C1	this study
UW6464	**+**	CCI	M2	2006	35	18	7-1-1-1-5-1-1	C1/17	5-7-3-3-2-3	1	C1	this study
UW6475	**+**	CCI	M2	2006	32	18	7-1-1-1-5-1-1	C1/17	5-7-3-3-2-3	1	C1	this study
UW6455	**+**	CCI	M2	2006	11	192	15-1-1-1-1-7-1	C1/17	5-7-3-3-1-2	159	C1	this study
UW6474	**+**	CCI	M2	2006	8	192	15-1-1-1-1-7-1	C1/17	5-7-3-3-1-2	159	C1	this study
UW6324	**+**	CCI	T	2005	20	203	15-1-1-1-1-20-1	C1/17	5-5-3-3-1-2	215	C1	this study
UW6337	**+**	CCI	T	2005	20	203	15-1-1-1-1-20-1	C1/17	5-5-3-3-1-2	215	C1	this study
UW5869	**+**	CCI	T	2004	6	279	1-5-1-1-1-7-1	C1/17	5-7-1-4-2-3	231	C1	this study
UW6321	**+**	CCI	T	2005	7	280	1-3-1-1-1-1-1	C1/17	5-7-3-3-2-3	1	C1	this study
UW6322	**+**	CCI	T	2005	22	282	7-1-1-1-5-20-1	C1/17	5-7-3-3-2-3	1	C1	this study
UW6323	**+**	CCI	T	2005	25	283	15-25-1-1-1-20-1	C1/17	5-7-3-3-1-2	159	C1	this study
UW6338	**+**	CCI	U	2005	21	203	15-1-1-1-1-20-1	C1/17	5-7-3-3-1-2	159	C1	this study
UW6343	**+**	B/CCI	U	2005	20	203	15-1-1-1-1-20-1	C1/17	5-7-3-3-1-2	159	C1	this study
UW6151	**+**	B/CCI	K2	2005	9	17	1-1-1-1-1-1-1	C1/17	5-7-3-3-2-3	1	C1	this study
UW6256	**+**	CCI	K2	2005	11	192	15-1-1-1-1-7-1	C1/17	5-7-3-3-1-2	159	C1	this study
UW6352	**+**	B/CCI	M3	2005	30	18	7-1-1-1-5-1-1	C1/17	5-7-3-3-2-3	1	C1	this study
UW6397	**+**	B/CCI	M3	2005	31	18	7-1-1-1-5-1-1	C1/17	5-7-3-3-2-3	1	C1	this study
UW6203	**+**	B/CCI	S1	2005	13	17	1-1-1-1-1-1-1	C1/17	5-7-3-3-2-3	1	C1	this study
UW6379	**+**	B/CCI	S1	2005	28	18	7-1-1-1-5-1-1	C1/17	5-7-3-3-2-3	1	C1	this study
UW6494	**+**	B/CCI	S1	2006	26	78	15-1-1-1-1-1-1	C1/17	5-7-3-3-1-2	159	C1	this study
UW6498	**+**	B/CCI	G2	2006	28	18	7-1-1-1-5-1-1	C1/17	5-7-3-3-2-2	7	C1	this study
UW6500	**+**	CCI	G2	2006	28	18	7-1-1-1-5-1-1	C1/17	5-7-3-3-2-2	7	C1	this study
UW6571	**+**	B/CCI	P	2006	27	78	15-1-1-1-1-1-1	C1/17	5-7-3-3-1-2	159	C1	this study
UW6589	**+**	CCI	W2	2006	6	17	1-1-1-1-1-1-1	C1/17	5-7-3-3-2-3	1	C1	[20]
UW6605	**+**	CCI	W2	2006	14	78	15-1-1-1-1-1-1	C1/17	5-7-3-3-1-3	12	C1	[20]
UW6511	**+**	B/CCI	B-S	2006	17	202	1-1-1-1-1-7-1	C1/17	5-7-3-3-2-2	7	C1	this study
UW6517	**+**	CCI	B-S	2006	17	202	1-1-1-1-1-7-1	C1/17	5-7-3-3-2-2	7	C1	this study
UW6528	**+**	CCI	B-S	2006	17	202	1-1-1-1-1-7-1	C1/17	5-7-3-3-2-2	7	C1	this study
UW6520	**+**	CCI	B-S	2006	5	314	43-5-1-1-12-7-1	C	5-7-1-4-2-3	231	C1	this study

**Group II**												

UW901	**+**	CCI	D	1996	15	117	9-1-1-1-1-1-1	C1/17	5-7-3-3-1-3	12	C1	[30]
UW931	**+**	CCI	G2	1996	15	117	9-1-1-1-1-1-1	C1/17	5-7-3-3-1-3	12	C1	[30]
UW1505	**+**	CCI	L4	1997	15	117	9-1-1-1-1-1-1	C1/17	5-7-3-3-1-3	12	C1	[30]
UW1806	**+**	CCI	B-F	1998	15	117	9-1-1-1-1-1-1	C1/17	5-7-3-3-1-3	12	C1	[30]
UW1822	**+**	CCI	E	1998	15	117	9-1-1-1-1-1-1	C1/17	5-7-3-3-1-3	12	C1	[30]
UW1823	**+**	CCI	B-K	1998	15	117	9-1-1-1-1-1-1	C1/17	5-7-3-3-1-3	12	C1	[30]
UW1827	**+**	CCI	H2	1998	15	117	9-1-1-1-1-1-1	C1/17	5-7-3-3-1-3	12	C1	[30]
UW2322	**+**	CCI	B-U	1999	15	117	9-1-1-1-1-1-1	C1/17	5-7-3-3-1-3	12	C1	[30]

**Group I**												

70/90	**+**	I/HS	B-C	1991	38	25	9-3-1-6-1-1-1	C/22	3-2-4-2-1-3	113	B	[31]
U200	**+**	I/HS	M4	1992	37	8	5-2-1-6-1-7-1	B/26	7-7-3-2-1-3	98	C	[31]
L283	**+**	I/HS	L3	1993	18	17	1-1-1-1-1-1-1	C1/17	5-5-3-3-2-3	163	C1	[31]
6011	**+**	I/HS	B-C	1993	36	189	3-2-1-9-1-20-5	A/5	7-4-3-1-1-1	238	A	[31]
18912	**+**	I/HS	P	1992	3	17	1-1-1-1-1-1-1	C1/17	5-7-3-3-2-3	1	C1	[31]
2513	**+**	I/HS	S2	1994	10	22	2-3-1-2-1-1-1	C/22	6-7-3-4-2-3	178	C1	[31]
UW786	**+**	CCI	N	1995	16	307	1-11-1-1-1-1-1	C1/17	5-7-1-4-2-3	231	C1	[31]
BM4147	**+**	R	Ref.	1986	n.d.	25	9-3-1-6-1-1-1	C/22	5-2-3-2-1-1	95	A	[40]

**Non-hospital origin**												

UW315	**+**	OS	-	1994	n.d.	n.d.		-	6-4-3-1-1-1	89	A	[31]
UW328	**+**	OS	-	1994	n.d.	n.d.		-	5-7-1-3-2-1	241	C1	[31]
UW440	**+**	OS	-	1994	n.d.	n.d.		-	6-4-3-1-1-1	89	A	[31]
UW2500	**-**	OS	-	1999	n.d.	n.d.		-	6-4-3-1-1-1	89	A	[32]
UW2508	**-**	OS	-	1999	n.d.	n.d.		-	6-4-1-3-1-2	242	B	[32]
UW2516	**-**	OS	-	1999	n.d.	n.d.		-	N-N-1-3-1-2	n.t.	-	[32]
UW2636	**-**	OS	-	1999	n.d.	n.d.		-	6-N-2-2-1-1	n.t.	-	[32]
UW2642	**-**	OS	-	1999	n.d.	n.d.		-	6-4-3-1-1-2	173	A	[32]
UW2647	**-**	OS	-	1999	n.d.	n.d.		-	6-4-3-1-1-1	89	A	[32]
UW2651	**-**	OS	-	1999	n.d.	n.d.		-	6-4-3-1-1-1	89	A	[32]
UW2655	**-**	OS	-	1999	n.d.	n.d.		-	5-N-1-3-1-1	n.t.	-	[32]
UW2712	**-**	OS	-	1999	n.d.	n.d.		-	3-6-1-4-1-3	243	C	[32]
UW2721	**-**	OS	-	1999	n.d.	n.d.		-	6-6-4-2-1-1	244	A	[32]
UW2726	**-**	OS	-	1999	n.d.	n.d.		-	5-2-4-3-1-2	245	B	[32]
UW2729	**-**	OS	-	1999	n.d.	n.d.		-	4-0-7-2-1-N	n.t.	-	[32]
UW2743	**-**	OS	-	1999	n.d.	n.d.		-	5-2-4-3-1-2	245	B	[32]
AW1	**+**	S	-	1991	n.d.	n.d.		-	5-4-1-3-3-2	122	B	[31]
AW2	**+**	S	-	1991	n.d.	n.d.		-	5-4-3-1-1-1	90	A	[31]
AW5	**+**	S	-	1991	n.d.	n.d.		-	5-4-3-1-1-1	90	A	[31]
AW9	**+**	S	-	1991	n.d.	n.d.		-	6-4-3-1-1-1	89	A	[31]
AW12	**+**	S	-	1991	n.d.	n.d.		-	3-4-4-2-1-2	103	B	[31]
UW4	**+**	F	-	1993	n.d.	n.d.		-	6-4-3-1-1-1	89	A	[31]
UW7	**+**	F	-	1993	n.d.	n.d.		-	6-4-3-1-1-2	173	A	[31]
UW53	**+**	M	-	1994	n.d.	n.d.		-	4-N-4-2-1-2	n.t.	-	[31]
UW68	**+**	F	-	1994	n.d.	n.d.		-	5-N-4-2-1-2	n.t.	-	[31]
UW261	**+**	M	-	1994	n.d.	n.d.		-	6-4-3-1-1-1	89	A	[31]
UW262	**+**	M	-	1994	n.d.	n.d.		-	6-4-3-1-1-1	89	A	[31]

Legend: Only one isolate per case/patient was allowed. Legend: CC, clonal complex, assigned according to references [14,16,41]. Origin: CCI, epidemic isolate/cluster of colonizations and infections (n ≥ 2); B, blood stream isolate; I/HS, from single infection or hospital patient stool sample; OS, outpatient stool sample; S, sewage isolate; F, poultry or pig farm; M, poultry meat or pork sample; R, reference isolate; *vanA*, *vanA *glycopeptide resistance genotype; VRE, vancomycin-resistant *E. faecium*; n.t., not typeable; n.d., not determined. ^1 ^N, no amplification of this locus; MLST scheme: *atpA*, *ddl*, *gdh*, *purK*, *gyd*, *pstS*, *adk*; MLVA scheme: VNTRs 1,2,7,8,9,10.

**Table 2:** Discriminatory indices for and congruence values between PFGE, MLST and MLVA for all 58 hospital *E. faecium*. [37–39]

	PFGE	MLST	MLVA	Discriminatory index	Confidence interval (95% CI)
PFGE		0.935	0.863	0.972	[0.951–0.992]
MLST	0.935		0.873	0.91	[0.877–0.943]
MLVA	0.863	0.873		0.842	[0.79–0.893]
